# The effectiveness and safety of acupoint herbal patching for allergic rhinitis: protocol for a systematic review and meta-analysis

**DOI:** 10.1186/s13643-024-02598-x

**Published:** 2024-07-06

**Authors:** Ningcong Xu, Claire Shuiqing Zhang, Xi Tan, Yunjie Lai, Yanfang Cen, Shiqing Zhou, Jiyan Xia, Yunying Li, Qiulan Luo

**Affiliations:** 1 The Second Clinical Medical College of Guangzhou University of Chinese Medicine, Airport Road 12, Baiyun District, Guangzhou, China; 2 China-Australia International Research Centre for Chinese Medicine, School of Health and Biomedical Sciences, RMIT University, Bundoora, VIC 3083 Australia; 3 Otorhinolaryngology Head and Neck Department, The Second Affiliated Hospital of Guangzhou University of Chinese Medicine, Yuexiu District, Dade Road 111, Guangzhou, China; 4 Otorhinolaryngology Head and Neck Department, Guangdong Provincial Hospital of Chinese Medicine, Yuexiu District, Guangzhou, China; 5 Foshan Hospital of Traditional Chinese Medicine, Chancheng District, Qinren Road 6, Foshan, China; 6 Community Health Service Center of Dagang Town, Nansha District, Tanzhou Boai Avenue 49, Guangzhou, China

**Keywords:** Allergic rhinitis, Acupoint herbal patching, Systematic review, Meta-analysis, Protocol

## Abstract

**Background:**

Allergic rhinitis (AR) is a common inflammatory disease of the nasal mucosa that is characterized by symptoms such as sneezing, nasal congestion, nasal itching, and rhinorrhoea. In recent years, acupoint herbal patching (AHP) therapy has gained a growing interest as a potential management option for AR. This systematic review and meta-analysis will evaluate the clinical research evidence on the effectiveness and safety of AHP as a treatment option for AR outside of the Sanfu or Sanjiu days (summer or winter solstice). The results of this review will provide up-to-date evidence-based guidance for healthcare providers and individuals seeking alternative treatments for AR.

**Methods:**

A comprehensive search of electronic databases (PubMed, Cochrane Central Register of Controlled Trials (CENTRAL), EMBASE, China National Knowledge Infrastructure (CNKI), CQVIP, Sino-Med, and Wanfang Databases) will be conducted from their inception to June 2023. The inclusion criteria will be limited to randomized controlled trials that evaluate the effectiveness or efficacy of non-Sanfu or non-Sanjiu AHP for AR. The primary outcome measure will be the total nasal symptom score. The methodological quality of included studies will be assessed using the Revised Cochrane risk-of-bias tool for randomized trials (RoB 2), and meta-analyses will be performed using RevMan (V.5.3) statistical software. The Grading of Recommendations Assessment, Development and Evaluation (GRADE) approach will be used to determine the certainty of evidence.

**Discussion:**

This systematic review and meta-analysis will provide valuable insights into the effectiveness and safety of non-Sanfu or non-Sanjiu AHP as a treatment option for AR. The study aims to produce a high-quality review by adhering to PRISMA-P guidelines and using clinical guideline recommended outcome measures. The results of this review may offer additional treatment options for AR patients who seek complementary and alternative therapies, and hold significant implications for future research in this field. Overall, this study has the potential to inform clinical practice and improve patient outcomes.

**Systematic review registration:**

PROSPERO CRD42022181322.

**Supplementary Information:**

The online version contains supplementary material available at 10.1186/s13643-024-02598-x.

## Strengths and limitations of this study

- To our knowledge, this will be the first systematic review and meta-analysis to investigate the efficacy and safety of non-Sanfu or non-Sanjiu acupoint herbal patching (AHP) as a treatment option for allergic rhinitis (AR). Previous systematic reviews have provided evidence supporting the use of Sanfu and Sanjiu AHP, which are administered during the summer or winter solstice, for the management of AR. The review will evaluate current available clinical research on non-Sanfu or non-Sanjiu AHP to benefit clinical practice.
- To ensure transparency and rigor in our systematic review and meta-analysis, we have registered our protocol with the International Prospective Register of Systematic Reviews (PROSPERO) (registration number [CRD42022181322]). We will follow the Preferred Reporting Items for Systematic Review and Meta-Analysis Protocols (PRISMA-P) guidelines to conduct the review and report the results. By adhering to these guidelines, we aim to produce a high-quality systematic review and meta-analysis that can provide valuable insights into the effectiveness and safety of non-Sanfu/non-Sanjiu AHP for AR.
- This systematic review will use clinical guideline recommended outcome measures, and provide results that can be directly translated into clinical practice.
- Limitation: the quality of the included studies may vary, which could impact the certainty of evidence, the diversity of comparative interventions may introduce complexity in data analysis and make it difficult to interpret the results in this meta-analysis.

## Background

### Allergic rhinitis: prevalence and burden

Allergic rhinitis (AR) is a prevalence inflammatory disease that occurs as a result of immunoglobulin E (IgE)-mediated hypersensitivity reactions of the nasal mucosa to allergens. The symptoms of allergic rhinitis include one or more of the following: sneezing, nasal congestion, nasal itch, and rhinorrhoea [1, 2]. The global prevalence of AR is estimated to be between 10 and 40% [3]. In China, the standardized prevalence of self-reported adult AR is 17.6% [4]. As one of the most common diseases affecting people of all ages, allergic rhinitis has become a significant concern worldwide [5–7]. It negatively impacts the quality of life (QoL) of many patients and affects their work or school performance [1, 4]. Consequently, AR imposes a significant economic burden, including direct costs of medical care and indirect costs associated with decreased work productivity [1, 8].

### Intervention for AR

Currently, there are four main treatment regimens for AR: patient education, allergen avoidance, pharmacotherapies, and allergen-specific immunotherapy [1, 8, 9]. Allergen avoidance is the most direct and effective method; however, to completely avoid common airborne allergens is not possible for most of the patients [10]. Pharmacotherapy includes intranasal steroids, oral / intranasal antihistamines, and oral leukotriene receptor antagonists [1, 8, 9]. While these pharmacotherapies provide satisfactory short-term symptom relief to most AR patients, they are often associated with side effects such as epistaxis, septal perforation, mucosal dryness, and sedation [9]. In addition, around 20% of AR patients experience symptoms that cannot be well-controlled by pharmacotherapies [2]. Allergen-specific immunotherapy is effective for long-term symptom control of AR, but it requires long treatment duration and is associated with high costs and the risk of severe allergic reactions [1, 9, 11].

It is worth noting that complementary and alternative therapies are often used in clinical practice for AR management. The clinical guideline published by the American Academy of Otolaryngology-Head and Neck Surgery (2015) acknowledged that there are certain evidence supporting the use of acupuncture for AR, but there is no recommendation regarding the use of herbal therapy [9].

### Purpose and rationale for this systematic review and *meta*-analysis

Acupoint herbal patching (AHP), also known as acupuncture point application, Tianjiu, acupoint herbal medicine patching, and acupoint herbal plaster, is a treatment method in which processed Chinese herbal preparations are applied directly to specific acupoints [12, 13]. It is generally believed that the therapeutic effects of AHP are produced by a combination of herbal infiltration absorption and acupoint stimulation [14]. AHP therapy was first introduced by a classical Chinese medicine book Zhang Shi Yi Tong published in the Qing dynasty (AD 1695) [15]. This therapy is considered an effective and convenient treatment for respiratory diseases including asthma and AR, and it is commonly used in the mainland of China and Taiwan [16, 17]. In recent years, clinical research has proved that AHP was effective for preventing asthma attacks [18, 19] and reducing AR symptoms [12, 13, 17, 20–22]. Several studies have reported that AHP can reduce serum total IgE levels [23], blood eosinophil counts [24], and serum IL-4 levels [23], and increase IFN-γ levels in patients with AR [23, 25]. AHP is usually implemented during the Sanfu or Sanjiu days (summer or winter solstice), namely Sanfu AHP or Sanjiu AHP [26]. Sanfu AHP or Sanjiu AHP has been suggested as an effective method for relieving AR nasal symptoms relief and preventing the reoccurrence of AR [20, 27].

Both Sanfu and Sanjiu days are only a short period of time (3 days in summer and 3 days in winter, respectively) in a year. It is inconvenient for AR patients to strictly arrange their AHP treatments during the specific Sanfu or Sanjiu days. More importantly, AR is a recurrent chronic airway disease; even during asymptomatic days, persistent inflammation is present at the mucosal level in patients with AR [28], highlighting the need for long-term continuous administration of a treatment during both the onset and remission stages of AR [29]. Applying AHP beyond the Sanfu or Sanjiu days may provide an effective and convenient therapy to AR patients. Although previous clinical trials and systematic reviews have proved Sanfu/Sanjiu AHP as an effective and safe for treating AR [12, 13, 16, 17], there has not been a systematic review that evaluates the effects of non-Sanfu/non-Sanjiu AHP for AR management. A recently published systematic review demonstrated that there was no significant difference between Sanfu AHP/Sanjiu AHP and non-Sanfu AHP/non-Sanjiu AHP, in terms of total effective rate and serum IgE; but this systematic review did not evaluate the treatment effects using standard outcome measures [30]. Therefore, we designed a systematic review focusing on non-Sanfu/non-Sanjiu AHP for AR by evaluating the most up-to-date clinical evidence using clinical guideline recommended outcome measures. This systematic review and meta-analysis aims to produce a high-quality evidence to evaluate the effectiveness and safety of non-Sanfu/non-Sanjiu AHP for AR, which will assist evidence-based clinical practice.

## Methods

### Protocol and registration

The protocol of this systematic review has been registered with PROSPERO (ID CRD42022181322), which can be accessed at https://www.crd.york.ac.uk/PROSPERO/. Detailed methods will be explained in this protocol, following the Preferred Reporting Items for Systematic Review and Meta-Analysis Protocols (PRISMA-P) checklist [31] (Additional file 1).

### Inclusion criteria

Only RCTs that evaluate the treatment effects of non-Sanfu AHP/non-Sanjiu AHP will be included in this review. Detailed inclusion criteria are:

#### Participants

Participants of any gender, age, ethnicity, or disease severity who have been diagnosed with AR according to specific criteria (such as diagnostic criteria recommended by any clinical guidelines) are eligible for inclusion in this study, regardless of the underlying cause.

#### Interventions

All types of AHP, regardless of the herbal regimen and acupoints selected, are eligible for inclusion. AHP was implemented during non-Sanfu and non-Sanjiu days. AHP was defined as sticking herbal patches on acupoints of the patient’s body. This type of AHP is an external therapy that prevents or treats conditions through the combined functions of herbal regimens and acupoints.

#### Comparisons

The control group received no treatment, placebo, or internationally recognized pharmacotherapy. Clinical trials using non-Sanfu/non-Sanjiu AHP combined with pharmacotherapy or immunotherapy compared with the same pharmacotherapy/immunotherapy will also be included in this review. The possibilities of the therapy combination group are listed as follows:

1. Non-Sanfu/non-Sanjiu AHP vs. no treatment.
2. Non-Sanfu/non-Sanjiu AHP vs. placebo or sham AHP
3. Non-Sanfu/non-Sanjiu AHP + pharmacotherapy/immunotherapy vs. the same pharmacotherapy/immunotherapy
4. Non-Sanfu/non-Sanjiu AHP + pharmacotherapy/immunotherapy vs. placebo or sham AHP + the same pharmacotherapy/immunotherapy

#### Outcome measures

We will use the total nasal symptom score (TNSS) as the primary outcome in this review. TNSS is the sum of four nasal symptom scores, namely stuffy nose, itchy nose, sneezing, and runny nose. It is usually used as a four-point scale: 0 for no symptoms, 1 for mild symptoms (symptoms present but not troublesome), 2 for moderate symptoms (troublesome symptoms but tolerable), and 3 for severe symptoms (intolerable symptoms with impairment of daily activities and/or sleep). The TNSS score ranges from 0 to 12, with a lower score indicating a milder symptom, and the minimal clinically important difference of TNSS is 0.55 point [32]. The secondary outcome measures will include the effective rate (a composite outcome measure that calculates the change in nasal symptom scores and clinical signs) [33, 34], AR symptom rating scale [32–34], visual analogue scale (VAS) [35], nasal signs score [33, 34], patients’ QoL using the Rhinoconjunctivitis Quality of Life Questionnaire (RQLQ) score [36] or the Medical Outcomes Survey Short Form 36 ( SF-36) [37], recurrence rate, serum IgE level, serum eosinophils (EOS) level, and adverse events.

### Search methods for the identification of studies

We will search the following electronic databases from their inception to June 2024: PubMed, the Cochrane Central Register of Controlled Trials (CENTRAL) in the Cochrane Library, EMBASE, China National Knowledge Infrastructure (CNKI), VIP Database, Sino-Med Database, and Wanfang Database. No restriction will be placed on the language of publication.

We will search ongoing trials from mainstream registries, including Current Controlled Trials (http://www.controlled-trials.com), the World Health Organization International Clinical Trials Registry Platform (WHO ICTRP; http://apps.who.int/trialsearch/), ClinicalTrials.gov trials registry (http://www.ClinicalTrials.gov), The Australian New Zealand Clinical Trials Registry (http://www.anzctr.org.au), and CentreWatch (http://www.centerwatch.com).

We will also manually review the reference lists of all full-text papers without language restrictions for additional relevant reports. Searches will be limited to randomized clinical trials, and a filter will be applied to limit manual searches. Details of the search strategy will be available from the authors upon request.

### Search strategy

The search strategy of PubMed is shown in box 1 (Additional file 2) as an example. This search strategy will be modified when applying to other databases.

### Study collection

All articles obtained from searching databases, grey literature, conference abstracts, and reference lists of relevant publications will be managed using EndNote X7 bibliographical software. Duplicated articles will be removed after verification.

### Data extraction

**Fig. 1:**
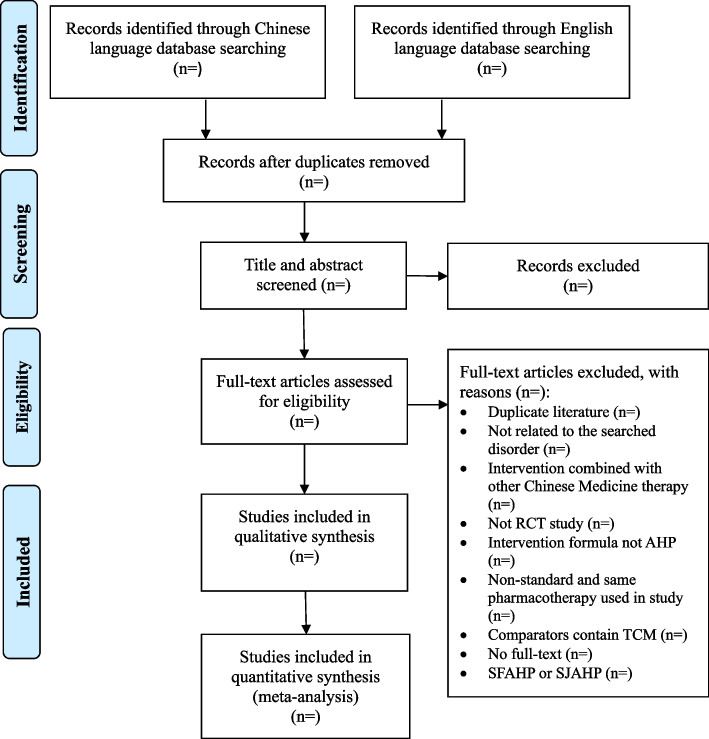
Flow diagram of the study design

Two reviewers (Ningcong Xu and Yanfang Cen) will independently screen all studies against the established inclusion criteria by checking their titles, abstracts, and full texts. The procedure of study selection is shown in the flow chart (Fig. 1). A standard data extraction form (Excel) will be used to extract information from included studies, and two reviewers (Ningcong Xu and Yunjie Lai) will independently extract data for the following items from the included studies and enter the data in a pre-defined Excel spreadsheet: first author, publication year, title, journal, sample size, arms, average age, courses of disease, interventions (preparations and ingredients of intervention, control intervention), acupoints, comparisons, duration of treatment, outcomes, methods of randomization, blinding, missing data, follow-up, and adverse events. Discrepancies arising during screening and data extraction will be resolved by discussing with a third reviewer (Jiyan Xia).

### Assessment of risk of *bias*

Two reviewers (Xi Tan and Shiqing Zhou) will independently assess the methodological quality of included studies using the Revised Cochrane risk-of-bias tool for randomized trials (RoB 2) (https://www.riskofbias.info/). In RoB 2, there are five domains of bias will be assessed: (1) bias arising from the randomization process, (2) bias due to deviations from intended intervention, (3) bias due to missing outcome data, (4) bias in measurement of the outcome, and (5) bias in selection of the reported result. Among these five domains, the bias in measurement of the outcome, the bias due to missing outcome data, and the bias in selection of the reported results are sensitive to the nature and handling of outcome measures. Therefore, the risk of bias assessment will be conducted on individual outcome measures. Each domain contains several signalling questions with five response options: Yes, Probably yes, Probably no, No, and No information. Based on the reviewer’s responses to the signalling questions, the risk of bias for each bias domain will be judged according to the algorithm, with suggested judgements as follows: “low risk”, “some concerns”, and “high risk”. An overall risk of bias will be given according to the assessment of the five domains. If the risk of bias in all domains is judged as “low risk”, the overall risk of bias will be considered “low risk”. If the risk of bias in any domain is judged as “some concerns”, and no domain is judged as “high risk”, the overall risk of bias will be considered “some concerns”. The overall risk of bias will be judged “high risk” if the risk of bias in one or more domains is judged as “high risk”. In cases of disagreement between reviewers, a third reviewer (Qiulan Luo) will be consulted to resolve the discrepancy.

### Measurement of treatment effect

Data analysis and synthesis will be conducted using RevMan (version 5.3) software. Dichotomous data will be presented as a risk ratio (RR) with corresponding 95% confidence intervals (CI), while continuous data will be expressed as mean differences (MD) with 95% CI. For studies reporting different scales for the same outcome measure, the standardized mean difference (SMD) will be employed. The meta-analysis results will be presented using forest plots, while the publication bias analysis of the results will be presented with the funnel plots. Heterogeneity will be assessed using the *χ*^2^ test and *I*^2^ test in RevMan (version 5.3). If the *I*^2^ value is less than 50%, a fixed-effect model will be used; otherwise, a random effects model will be applied. A two-sided *p*-value will be calculated for each meta-analysis with a significance level of *α* = 0.05.

### Dealing with missing data

In order to address missing or incomplete data within the study, efforts will be made to contact the primary or corresponding author of the original article via email or phone call for elucidation. In the absence of a response, such a study will be excluded from our meta-analysis.

### Assessment of publication *bias*

If the meta-analysis includes more than 10 studies, funnel plots will be generated, and Egger’s test will be performed using Stata14 to identify potential reporting bias. If the two sides of the funnel plot are asymmetric, this suggests a high possibility of publication bias, and Egger’s test will be used to confirm this. A *p*-value of less than 0.05 indicates the presence of publication bias, whereas a *p*-value of greater than 0.05 suggests the absence of such bias, as indicated by the results of the funnel plot.

### Subgroup analysis

Where data is available, subgroup analysis will be performed based on the age of subjects, different types of AHP interventions, types of treatment used in the control groups (such as pharmacotherapy medicines and immunotherapy), severity of disease, treatment duration, and acupoint used for non-Sanfu/non-Sanjiu AHP.

### Sensitivity analysis

If necessary, sensitivity analysis will be conducted in accordance with the recommendations outlined in the Cochrane Handbook to evaluate the reliability and robustness of the results obtained during the review process. Factors such as sample size and methodological quality of the studies included in the analysis will be taken into account for sensitivity analysis.

### Certainty in evidence

Two reviewers (Ningcong Xu and Qiulan Luo) will use the Grading of Recommendations Assessment, Development and Evaluation (GRADE) approach [38] to summarize and evaluate the certainty in evidence. There are five factors that GRADE may reduce the certainty of evidence in interventional systematic reviews: bias risk, inconsistency, inaccuracy, indirectness, and publication bias. The above five factors will be evaluated using the GRADEpro software. The certainty of evidence will be divided into the following four levels: high, medium, low, and very low. These different levels of certainty represent the strength of the evidence. In cases where there is disagreement between reviewers, a third reviewer (Claire Shuiqing Zhang) will be consulted to resolve the disagreement.

### Ethics and dissemination

As patients or private data will not be collected, there are no ethical considerations and no need for ethical approval. The results will be published in a peer-reviewed journal.

## Discussion

Allergic rhinitis (AR) is a globally prevalent condition that significantly impacts patients’ quality of life and imposes a substantial economic burden on society. Due to persistent inflammation caused by AR, it is widely acknowledged that patients with AR require treatments that are safe for long-term use, clinically effective, easy to administer, and produce anti-inflammatory effects [29]. Sanfu AHP or Sanjiu AHP is time-honored complementary and alternative therapies commonly used in clinical practice. Previous clinical trials and systematic review have reported that these therapies are beneficial for AR [12, 13, 17, 20–22], suggesting they are effective methods for relieving AR nasal symptoms and preventing the reoccurrence [16, 17]. The prevailing hypothesis posits AHP may generate specific therapeutic effects through a combination of herbal infiltration absorption and acupoint stimulation [14, 39]. However, many AR patients are unable to receive these treatments during the specific Sanfu or Sanjiu days or prefer to apply such treatments beyond those days. AHP is now available in most community hospitals/clinics in China, as it is a non-invasive, easy-to-administer, and safe therapy. Patients can purchase pre-made herbal powder mixtures and apply the herbal patches themselves, following clear instructions. Providing rigorous research evidence to support the application of AHP in non-Sanfu/non-Sanjiu days will increase the accessibility of AHP, and encourage AR patients to receive these treatments anytime when needed.

This systematic review and meta-analysis will assess the latest clinical evidence using recommended outcome measures and follow the PRISMA-P guidelines to provide high-quality evidence on non-Sanfu/non-Sanjiu AHP treatments to support evidence-based clinical practice. The findings of this review may offer additional treatment options for AR patients seeking complementary and alternative therapies, identifying knowledge gaps and having significant implications for future research. In conclusion, this study has the potential to inform clinical practice and improve patient outcomes.

It should be acknowledged that there are certain limitations in this study. Firstly, the variability in the methodological quality of the included studies, which can affect the certainty of the evidence obtained. Secondly, the diversity of comparative interventions (no treatment, placebo, pharmacotherapy, etc.) may introduce complexity in data analysis and make it difficult to interpret the results in this meta-analysis.

### Supplementary Information

Additional file 1: PRISMA-P checklist

Additional file 2: The search strategy of PubMed

## Data Availability

Not applicable.

## Abbreviations

AR: Allergic rhinitis
AHP: Acupoint herbal patching
CENTRAL: Cochrane Central Register of Controlled Trials
CNKI: China National Knowledge Infrastructure
IgE: Immunoglobulin E
QoL: Quality of life
PRISMA-P: Preferred Reporting Items for Systematic Review and Meta-Analysis Protocols
TNSS: Total nasal symptom score
VAS: Visual analogue scale
RQLQ: Rhinoconjunctivitis Quality of Life Questionnaire
SF-36: Survey Short Form 36
EOS: Eosinophils
RoB 2: Revised Cochrane risk-of-bias tool for randomized trials
RR: Risk ratio
CI: Confidence intervals
MD: Mean differences
SMD: Standardized mean difference
GRADE: Grading of Recommendations Assessment, Development and Evaluation
