# Supplementary material for: The effectiveness and safety of acupoint herbal patching for allergic rhinitis: protocol for a systematic review and meta-analysis
Source: Syst Rev. 2024 Jul 6;13:172. doi: 10.1186/s13643-024-02598-x (PMC11227191; doi:10.1186/s13643-024-02598-x)
Supplement: Supplementary file 2 — Additional file 2: The search strategy of PubMed [file 13643_2024_2598_MOESM2_ESM.docx]

**Box 1**: Pubmed will be searched until June 2024.

| Search number | Query |
| --- | --- |
| #1 | "Rhinitis, Allergic"[Mesh] |
| #2 | ((Allergic Rhinitides[Title/Abstract]) OR (Rhinitides, Allergic[Title/Abstract])) OR (Allergic Rhinitis[Title/Abstract]) |
| #3 | #1 OR #2 |
| #4 | (((((((((Chinese herbal patch[Title/Abstract]) OR (Tianjiu[Title/Abstract])) OR (acupoint application therapy[Title/Abstract]))) OR (Acupoint Herbal Patching[Title/Abstract])) OR (Herbal Point-Patch[Title/Abstract])) OR (Acupoint herbal plaster[Title/Abstract])) OR (acupuncture point application[Title/Abstract])) OR (acupoint Chinese Medicine patching[Title/Abstract])) OR (Chinese Medicine Acupoint Application[Title/Abstract]) |
| #5 | randomized controlled trial [Publication Type] OR (randomized [Title/Abstract] AND controlled [Title/Abstract] AND trial [Title/Abstract]) |
| #6 | #3 AND #4 AND #5 |
